# Smart drug delivery systems to overcome drug resistance in cancer immunotherapy

**DOI:** 10.20892/j.issn.2095-3941.2023.0009

**Published:** 2023-05-02

**Authors:** Wenzhe Yi, Dan Yan, Dangge Wang, Yaping Li

**Affiliations:** 1 State Key Laboratory of Drug Research & Center of Pharmaceutics, Shanghai Institute of Materia Medica, Chinese Academy of Sciences, Shanghai 201203, China; 2 University of Chinese Academy of Sciences, Beijing 100049, China; 3 Department of Pharmaceutics, School of Pharmacy, Nanjing Medical University, Nanjing 211116, China; 4 Yantai Key Laboratory of Nanomedicine & Advanced Preparations, Yantai Institute of Materia Medica, Yantai 264000, China; 5 Shandong Laboratory of Yantai Drug Discovery, Bohai Rim Advanced Research Institute for Drug Discovery, Yantai 264000, China

**Keywords:** Cancer immunotherapy, drug resistance, smart drug delivery system, immunosuppressive microenvironment, immune cell

## Abstract

Cancer immunotherapy, a therapeutic approach that inhibits tumors by activating or strengthening anti-tumor immunity, is currently an important clinical strategy for cancer treatment; however, tumors can develop drug resistance to immune surveillance, resulting in poor response rates and low therapeutic efficacy. In addition, changes in genes and signaling pathways in tumor cells prevent susceptibility to immunotherapeutic agents. Furthermore, tumors create an immunosuppressive microenvironment *via* immunosuppressive cells and secrete molecules that hinder immune cell and immune modulator infiltration or induce immune cell malfunction. To address these challenges, smart drug delivery systems (SDDSs) have been developed to overcome tumor cell resistance to immunomodulators, restore or boost immune cell activity, and magnify immune responses. To combat resistance to small molecules and monoclonal antibodies, SDDSs are used to co-deliver numerous therapeutic agents to tumor cells or immunosuppressive cells, thus increasing the drug concentration at the target site and improving efficacy. Herein, we discuss how SDDSs overcome drug resistance during cancer immunotherapy, with a focus on recent SDDS advances in thwarting drug resistance in immunotherapy by combining immunogenic cell death with immunotherapy and reversing the tumor immunosuppressive microenvironment. SDDSs that modulate the interferon signaling pathway and improve the efficacy of cell therapies are also presented. Finally, we discuss potential future SDDS perspectives in overcoming drug resistance in cancer immunotherapy. We believe that this review will contribute to the rational design of SDDSs and development of novel techniques to overcome immunotherapy resistance.

## Introduction

Cancer immunotherapy has become a major area of research in cancer treatment during the last decade owing to the documented extraordinary success in treating or managing tumors^1–4^. Cancer immunotherapy is a treatment strategy to keep cancer under control and eliminate cancer by reactivating and maintaining the immune system to identify and eliminate tumor cells. Cancer immunotherapy includes the use of cancer vaccines, cell therapy, immune checkpoint inhibitors, therapeutic antibodies, and small-molecule inhibitors^5^; however, the clinical responsiveness to immunotherapy varies greatly. Specifically, immune checkpoint blockade (ICB) has a clinical response rate of < 30%^6^. This finding is primarily a consequence of drug resistance to the immune system. Tumors have a complex genetic network that allows tumors to develop resistance to drugs that are detrimental to growth. For example, tumor cells overexpress programmed cell death protein 1 ligand (PD-L1), which inhibits cytotoxic T lymphocyte (CTL) activity by interacting with programmed cell death protein 1 (PD-1) on the T cell surface^7^. Indoleamine 2,3-dioxygenase 1 (IDO-1), which is overexpressed in tumor cells, reduces CTL function by increasing the conversion of tryptophan-to-kynurenine^8^. Mutations in gene expression or epigenetic changes result in decreased drug uptake, a greater efflux by tumor cells, or changes in drug target structure^9^. Furthermore, tumors have a heterogeneous metabolic regulatory network that maintains a complex heterogeneous immunosuppressive microenvironment by producing abnormal metabolites and recruiting various cells^10^. It is difficult for monoclonal antibodies and immune cells to overcome this barrier or become inactivated in response to numerous immunosuppressive cells or metabolites in the immunosuppressive microenvironment. These factors render tumor cells resistant to existing immunotherapies. Therefore, developing innovative techniques to overcome drug resistance is critical for enhancing cancer immunotherapy.

With the rapid advances in life sciences and nanotechnology, smart drug delivery systems (SDDSs) are under development for cancer immunotherapy^11^. SDDSs accurately deliver and release therapeutic agents to disease target sites (precise delivery and intelligent drug release) in response to specific stimuli by altering the structural functionalized components. For example, use of the acid-sensitive linker, maleic acid amide, to construct SDDSs has been shown to cause stimulus-activated disintegration in a tumor acid environment^12^. Matrix metalloproteinase-2 (MMP-2)- and MMP-9-sensitive materials respond to the tumor microenvironment by overexpressing MMP-2 and MMP-9^13^. Furthermore, reactive oxygen species (ROS)-responsive materials have been synthesized using thione linkers or boronic acid-containing adducts^14^. Moreover, some materials with external stimuli-sensitive elements, such as light, electricity, magnetic fields, and temperature, have demonstrated improved targeted drug delivery^15–17^. Owing to these reports, SDDSs are thought to be a promising approach to overcome tumor drug resistance and enhance cancer immunotherapy^18^. SDDSs efficiently target tumor cells by passive or active targeting, enriching drug accumulation, triggering tumor cell death, boosting tumor antigen exposure, and improving immune recognition^19^. In contrast, SDDSs target immune cells by altering the functional phenotype of immune cells, causing immune cells to evolve in an anti-tumor direction, and enhancing immune system function^20^.

**Figure 1:**
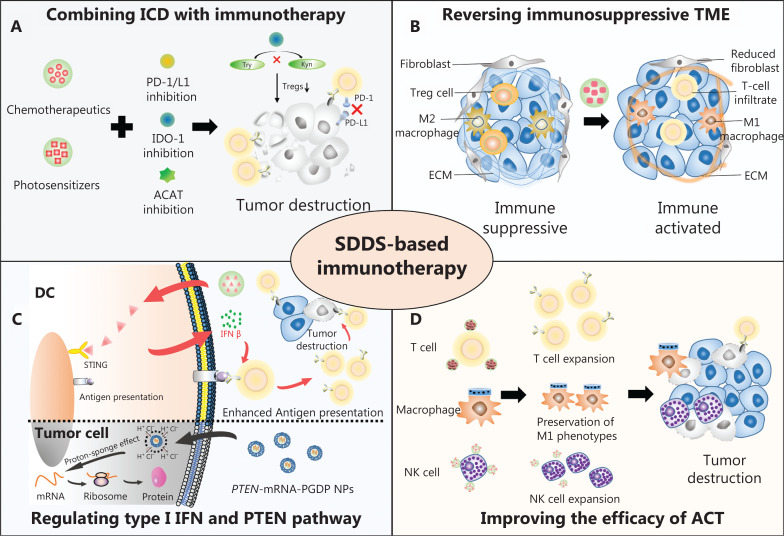
Schematic illustration of smart drug delivery systems (SDDSs) for overcoming drug resistance in cancer immunotherapy. (A) SDDSs deliver immunogenic cell death (ICD) inducers combined with immune checkpoint inhibitors, Indoleamine 2,3-dioxygenase 1 (IDO-1) inhibitors, and cholesterol metabolism regulators to tumor cells to enhance the killing effect of T cells on tumor cells. (B) SDDSs targeting immunosuppressive cells and extracellular matrix (ECM) reverse the immunosuppressive tumor microenvironment (TME), increase T cell infiltration, and reprogram M2 type macrophages to M1 type macrophages. (C) Upper part: SDDSs that modulate type I IFN signaling pathway enhance dendritic cell (DC) antigen presentation to enhance T cell killing of tumor cells; lower part: SDDSs that deliver *PTEN*-mRNA to tumor cells reverse PTEN expression to reverse immunotherapy resistance. (D) SDDSs combined with CAR-T-, NK cell-, and macrophage-based adoptive cell therapy (ACT) induce robust immune responses. ACAT, cholesterol esterase; Try, tryptophan; Kyn, kynurenine.

Several reviews have highlighted the progress of SDDS research for cancer immunotherapy in recent years, thus providing inspiring and prospective insight into the design of these delivery systems for cancer immunotherapy^21–24^; however, these reports did not discuss the application of SDDSs in overcoming drug resistance in cancer immunotherapy. Because drug resistance is currently one of the most significant issues in immunotherapy, it is imperative to discuss the use of SDDSs in immunotherapy to overcome drug resistance. Summarizing current SDDS evidence for overcoming drug resistance in cancer immunotherapy will also assist researchers in developing more efficient drug carriers and promote the use in immunotherapy. To this end, we have outlined the current mechanisms of drug resistance in cancer immunotherapy. Then, we focused on the mechanisms underlying SDDSs and applications of SDDSs from the perspective of overcoming drug resistance. Lastly, we discussed the existing obstacles and provide future prospective for the use of SDDSs in overcoming drug resistance in cancer immunotherapy (**Figure 1**).

## Mechanisms underlying drug resistance in cancer immunotherapy

There are three common clinical forms of resistance to cancer immunotherapy, as follows: (1) primary resistance, in which the tumor does not respond to immunotherapy from the beginning; (2) adaptive immune resistance, in which the tumor is recognized by the immune system but the tumor is protected by adapting to the immune attack; and (3) acquired resistance, in which the tumor initially responds to immunotherapy but becomes resistant to the drug after a period of treatment and begins to relapse^25^. Patients who have primary resistance to ICB do not respond to the initial therapy. The most straightforward reason why a tumor does not respond to immune checkpoint therapy or adoptive cell therapy (ACT) is a lack of recognition by T cells due to an absence of tumor antigens. Alternatively, patients develop adaptive resistance if the cancer cells have tumor antigens but develop mechanisms to avoid antigen presentation on the major histocompatibility complex (MHC)-restricted surface due to alterations in the antigen-presenting machinery. Patients who develop acquired resistance initially have an objective response to ICB therapy with anti-CTLA-4 or anti-PD-1 will relapse over time, even despite receiving continued therapy. With the advances in immunology and cell biology, researchers have recognized that drug resistance to cancer immunotherapy, whether primary, adaptive, or acquired, is a continual dynamic evolutionary process that is mediated by tumor cells and the tumor microenvironment (TME)^18^. Given the intricacy and diversity of tumor immunotherapy resistance mechanisms, we have categorized the mechanisms as follows: (1) tumor cell-intrinsic mechanisms rely on the baseline expression of resistance driver proteins and mutations in tumor cells; and (2) tumor cell-extrinsic mechanisms block cell death pathways and enhance survival behaviors and characteristics *via* dynamic interactions between the tumor and the TME.

### Tumor cell intrinsic mechanisms

Intrinsic cellular resistance mechanisms refer to the expression or inhibition of genes or pathways in tumor cells that limit immune cell infiltration or suppress the immunologic TME. These processes may exist intrinsically in tumor cells or emerge during tumor progression to influence drug uptake and T cell activation, thus preventing tumor death by the immune system^26^. Several inherent processes have recently been identified, as follows: (1) low drug uptake and high drug efflux; (2) target mutation; (3) low tumor antigenicity; (4) surface downregulation of surface MHC; (5) dual role of interferon (IFN) signaling; and (6) regulation of oncogenic signaling.

A drug must reach the target site at an adequate concentration to elicit a therapeutic effect. When a drug fails to attain the concentration required for therapy within a tumor cell, the drug fails to destroy the tumor, which may be due to drug entry failure or drug excretion. One of the most common issues pertaining to immunotherapy, is multidrug resistance gene amplification^27^. In this case, a gene encodes a transmembrane protein that prevents drug entry into the cell while excreting the drug. Multidrug resistance protein 1 (MDR1), multidrug resistance-associated protein 1 (MRP1), and breast cancer resistance protein (BCRP) are the three most frequently studied transporter proteins firmly linked to drug resistance in numerous cancers^28–30^. All of these transporter proteins have broad basal specificity and excrete foreign chemicals from the cell, thus allowing cancer cells to develop drug resistance. Drug resistance reduces tumor antigen production, which reduces tumor recognition and killing by the immune system, and affects the efficacy of immunotherapy following chemoresistance. The current therapeutic strategy is to use combinations of treatment agents to halt the process of tumor drug resistance, thus prolonging patient survival^31^. When the drug target changes, the efficacy is reduced, eventually leading to drug resistance in tumor cells^32^. For example, several anti-cancer medications inhibit topoisomerase II, resulting in DNA damage, inhibition of DNA synthesis, and the end of the mitotic process. Further, cancer cells become resistant to topoisomerase II through a variety of mechanisms^33^ due to mutations in the topoisomerase II gene. In addition, human epidermal growth factor receptor 2 (HER2), a tyrosine kinase receptor of the EGFR family, is overexpressed in 30% of patients with breast cancer, and resistance can develop following long-term use of inhibitors targeting this receptor^34^.

Decreasing tumor antigenicity and neoantigen production result in loss or diminished T cell recognition^35^. For example, long-stranded non-coding RNA (lncRNA) expression promotes degradation of antigenic peptide loading complexes (PLCs) and intrinsic tumor suppressors (Rb and p53) in triple-negative breast cancer (TNBC), resulting in antigenic downregulation and intrinsic tumor suppression^36^. Several studies have shown that tumor neoantigens are useful at inducing anti-cancer immune responses and exerting effectiveness in immunosuppressive malignancies^37,38^. Specialized T lymphocytes targeting neoantigens have been shown to proliferate and gain anti-cancer activity in response to ICB in a mouse sarcoma model^39^. Therefore, neoantigen-based cancer vaccines are projected to be an effective method for combating drug-resistant cancers^40^.

Tumor cells escape T cell killing by downregulating the surface MHC I pathway^41^. IFN is known to stimulate or enhance MHC I antigen presentation^42^, whereas deficiencies in antigen processing can impair MHC I surface expression. For example, changes in proteasome subunits or transporter proteins (TAPs), deletion of the 2-microglobulin B2M gene, or secondary changes in the structure of human leukocyte antigen (HLA)-1 class molecules all contribute to decreased MHC I production in tumor cells^43–45^. Developing targeted therapeutic strategies to address these alterations is expected to maintain MHC expression on the tumor surface, thereby improving the tumor-killing effect of T cells^46^.

IFNs are pleiotropic cytokines that have a key role in the coordination of tumor-immune system interactions^47^. IFNs inhibit tumor growth, promote tumor cell apoptosis, and increase immune cell activity^48^; however, IFNs also induce immune evasion by upregulating multiple immune checkpoints^49^. For example, T-cell responses against tumor antigens lead to IFN-γ expression in the TME, which activates Janus kinase (JAK)–signal transducer and activator of transcription (STAT) signaling, resulting in PD-L1 expression^50^. Induction of PD-L1 expression can be prevented by disrupting the tumor cell response to IFNγ signaling, rendering PD-1-PD-L1 blocking ineffective; however, this strategy represents a mechanism of resistance not only to the immune checkpoint, but also to anti-tumor immunity. Stronger anti-tumor effects are expected when ICB is combined with immunotherapy targeting IFNs^51^.

Oncogenic signaling pathways may be related to drug resistance at many stages of cancer development, including tumor initiation, growth, invasion, and metastasis. For example, activation of the WNT/β-catenin signaling pathway in tumor cells prevents T cells from entering into the TME and reduces the number of CD103^+^ dendritic cells (DCs)^52^. The mitogen-activated protein kinase (MAPK) signaling pathway contributes to cancer immune evasion by increasing expression of immunomodulatory cytokines (IL-6 and IL-10)^53^. Phosphatase and tensin homolog deleted on chromosome 10 (PTEN) protein inactivation mutations increase tumor cell resistance to cytotoxic T cells^54^. It is expected that therapies that specifically target these signaling pathways will address resistance in tumor immunotherapy.

### Tumor cell extrinsic mechanisms

Cell extrinsic mechanisms include regulatory T cells (Tregs), myeloid-derived suppressor cells (MDSCs), tumor-associated macrophages (TAMs), tumor-associated neutrophils (TANs), aberrant physiologic indicators, and suppressive immune checkpoints, all of which contribute to tumor immunotherapy resistance^55,56^.

Cancer cells secrete chemokines and cytokines that recruit MDSCs, TAMs, Treg, TANs, and other immunosuppressive cells to primary and secondary tumor sites in primary tumors^57^. These cells directly block the cytotoxic function of CD8^+^ T and NK cells, resulting in tumor cells that escape immune system destruction. Tregs, for example, suppress effector T cell responses through the secretion of suppressor cytokines, such as IL-10, IL-35, and TGF-β, as well as direct cellular interaction^58^. MDSCs regulate the immune system in a variety of ways, including the release of anti-inflammatory and suppressive cytokines (IL-10, TGF-β, and ROS), inducible nitric oxide synthase (iNOS) and Arg-1 expression, immune checkpoint expression, and synergy with other immune cells, such as Th17 and Tregs^59,60^. TAMs express PD-L1 and release IL-10^61^. TAMs also promote tumor growth by secreting MMPs, scraping the basement membrane, remodeling epithelial cell motility, releasing VEGF to stimulate angiogenesis, and recruiting Tregs and MDSCs^62^. Therefore, preventing tumor immune escape by targeting immunosuppressive cells at various stages of cancer growth could be a potential immunotherapeutic technique^63^.

It has been demonstrated that biochemical reactions in the TME cause significant metabolic abnormalities, resulting in disparities between the TME and normal tissues^64^. For example, an acidic environment (pH 6.2–6.9), overexpression of numerous enzymes, insufficient oxygen supply, and high glutathione (GSH) and ATP concentrations are some of the associated factors^65^. Additionally, aberrant metabolites produced by mutant tumor cells have been shown to influence effector T cell activity^66^. These signals can cause anti-tumor immune cells to stop functioning or increase the development of immunosuppressive receptors (e.g., PD-1, TIM-3, and LAG-3) on the surface of effector cells, thus boosting immunotherapy resistance^67,68^. Consequently, the development of targeted medicines or TME-responsive drug delivery systems for these abnormal physiologic indicators is predicted to reverse unfavorable TME conditions and boost or restore effector cell activity, thus overcoming immunotherapy resistance.

## SDDSs for overcoming drug resistance in cancer immunotherapy

SDDSs provide precise, targeted drug delivery and release in response to certain stimuli, thus potentially overcoming drug resistance in cancer immunotherapy. Current SDDSs utilize the following strategies to overcome drug resistance, as follows: (1) combining immunogenic cell death (ICD) with cancer immunotherapy; (2) reversing the tumor immunosuppressive microenvironment; (3) regulating IFN and PTEN signaling pathway; and (4) enhancing the efficacy of ACT. The rationale and recent research results for the different strategies are discussed below.

### SDDSs for inducing ICD in combined cancer immunotherapy

#### SDDS-based ICD

ICD is a mechanism by which tumor cells control the anti-tumor immune response in response to external stimuli^69^. ICD is recognized for its capacity to increase tumor antigen exposure, induce the release of tumor cell components, stimulate DC maturation, and activate adaptive antitumor immunity by activating T cells^70^. Currently, ICD inducers have been discovered in chemotherapeutic drugs, such as mitoxantrone and oxaliplatin^71^. ICD inducers are promising improved therapeutic applications due to anti-tumor cytotoxicity and activation of anti-cancer immunity; however, because of tumor efflux, the drug dose not attain a therapeutic concentration and the success rates of these agents in clinical practice are poor. SDDSs actively or passively target tumor tissues and deliver large amounts of ICD inducers to tumor cells, which increases the intracellular drug concentrations and efficiently induces ICD, resulting in increased DC maturation and CTL activation^72,73^. Li et al.^74^ proposed a dual endoplasmic reticulum (ER)-targeting strategy for photodynamic therapy (PDT) and photothermal therapy (PTT). ER-targeted pardaxin (FAL) peptide-modified indocyanine green (ICG)-conjugated hollow gold nanospheres (FAL-ICG-HAuNS) and hemoglobin (Hb) liposomes were used in this system (FAL-Hb liposomes). Under near-infrared (NIR) light irradiation, this nanosystem generated ICD and calreticulin (CRT) exposure, which served as an “eat-me” signal to enhance antigen presentation by DCs. This outcome triggered a cascade of immunologic responses, including CD8^+^ T cell growth and the release of cytotoxic cytokines. ER-targeted SDDS selectively regulates ER stress, thereby providing a controlled method for ICD-mediated tumor immunotherapy.

#### Combining ICD inducer with IDO-1 inhibitors

**Figure 2:**
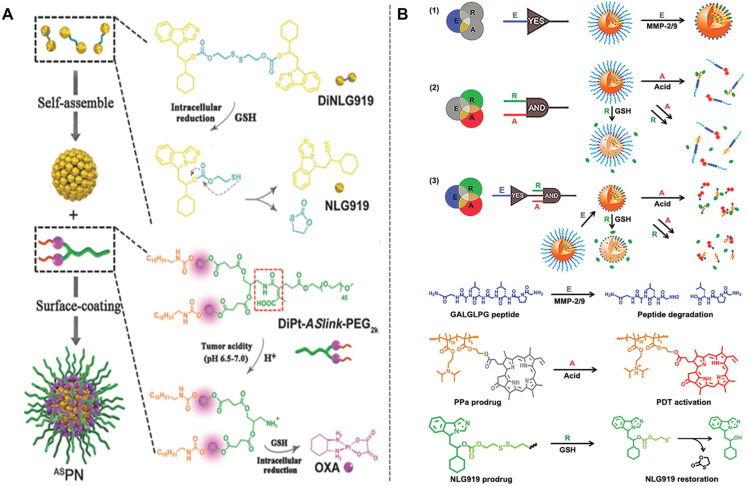
SDDSs combining ICD inducer with IDO-1 inhibitors. (A) Self-assembly procedure of TME-activated prodrug nanoparticles (BCPN), and chemical structure of DiNLG919 and DiPt-*ASlink*-PEG_2k_ prodrugs. Reproduced with permission from reference^77^. Copyright 2018 Wiley-VCH. (B) Construction of Boolean logic prodrug nanoparticles (BLPNs) for combination immunotherapy of cancer: (1) the E-YES-gate for MMP-2/9-triggered cleavage of the PEG coronary cleavage; (2) parallel-linkd A- and R-AND gates for acid-activated and GSH-triggered release of NLG919, respectively; and (3) higher-order BLPNs generated by hierarchical integration of E-YES and A/R-AND gates for codelivery of dual immune modulators and co-immunotherapy. Reproduced with permission from reference^79^. Copyright 2020 Wiley-VCH.

IDO-1 overexpression in tumor cells stimulates Tregs, while reducing effector T and NK cell activities. IDO-1 drugs inhibit tryptophan breakdown and reverses tumor cell suppression of T lymphocytes^75^; however, because IDO-1 inhibitors do not have a significant role in tumor immunotherapy, IDO-1 inhibitor use alone frequently fails to achieve therapeutic efficacy, similar to the combination with PD-1 checkpoint inhibitors for melanoma^76^. This finding could be attributed to limited T cell infiltration in tumor tissues and inhibition of anti-tumor immune activation. Combining IDO-1 inhibitors with ICD inducers using a SDDS may boost the immunotherapeutic efficacy of IDO-1 inhibitors. Feng et al.^77^ designed a TME-activated prodrug nanoparticle (BCPN) to achieve antitumor immunotherapy by synergistically inducing ICD and IDO-1 inhibition (**Figure 2A**). In both acidic and reducing TMEs, BCPN released oxaliplatin (OXA), while GSH activated the IDO-1 inhibitor prodrug, NLG919. Activated OXA increased CTL aggregation in cancer cells by activating ICD, while NLG919 inhibited IDO-1-mediated immunosuppression. To achieve spatiotemporal control of tumor aggregation and deep penetration of nanoparticles, Feng et al.^77^ also developed light-induced nanoparticles (LINCs) to control tissue penetration and drug release of these carriers by NIR, while inducing ICD and IDO-1 inhibition to improve tumor immunotherapy^78^. To more accurately adjust the cascade release of nanodrugs in TME, Hou et al.^79^ reported boolean logic prodrug nanoparticles (BLPNs) for co-delivery of pheophorbide A (PPa) and NLG919 (**Figure 2B**). As a biocomputational platform, BLPNs are activated cascade-after-cascade in response to various inputs. First, the enzymatic breakdown of the peptide sequence, GALGLPG (GG), in the TME by MMP-2 and MMP-9 overexpression permits tumor-specific accumulation and retention of BLPNs. Second, in the presence of GSH, the disulfide bond dissolves, thus releasing NLG919. Finally, 2-(diisopropylamino) ethyl methacrylate (DPA) decomposes to liberate PPa. The integration of various stimulatory signals into one chain can result in tumor-specific distribution and activation of BLPNs, thereby opening up new avenues for overcoming drug resistance in cancer immunotherapy.

#### Combining ICD inducers with ICB inhibitors

Although ICD inducers activate the immune system, the ICD inducer effectiveness is restricted by tumor resistance, especially due to the existence of immune checkpoints. One of the most important immune checkpoints for CTL activity suppression is the PD-1/PD-L1 pathway. Some PD-1/PD-L1 checkpoint inhibitors are currently available commercially; however, due to tumor heterogeneity and tumor-intrinsic resistance mechanisms, such as altered IFN signaling pathways, increased mutations, defective antigen presentation, and drug-resistant gene expression, the clinical response rate of PD-1/PD-L1 therapies is approximately 30%^80^. Therefore, several SDDS strategies have been created to deliver PD-1/PD-L1 antibodies^6^. Using SDDSs to co-deliver ICD inducers and PD-1/PD-L1 antibodies not only activates anti-tumor immunity but also prevents immunosuppression, which is a promising technique for tumor immunotherapy.

**Figure 3:**
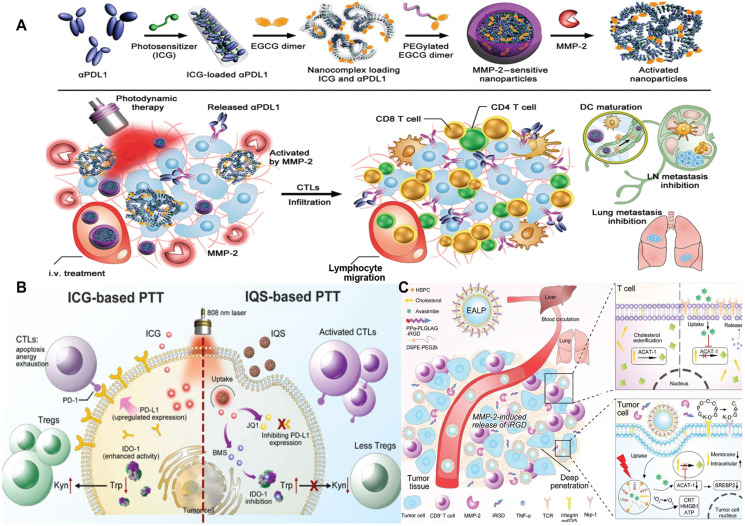
SDDSs combining ICD inducers with ICB inhibitors or cholesterol metabolism regulators. (A) Schematic illustration of MMP-2-sensitive aPDL1/ICG-based nanoparticle (S-aPDL1/ICG@NP)–mediated combined ICB and PDT. S-aPDL1/ICG@NP are activated in the TME with sustained release of aPDL1. Under NIR laser irradiation, ICG-mediated PDT induce anti-tumor immunity and promote the intratumoral infiltration of CTLs. i.v., intravenous. Reproduced with permission from reference^81^. Copyright 2019 American Association for the Advancement of Science. (B) ICG-based PTT upregulated PD-L1 and IDO-1 expression, triggered CTLs apoptosis and exhaustion by PD-1/PD-L1 interactions, and recruited Tregs by accumulating Kyn. When treated with IQS, dual blockade of immunosuppressive pathways by JQ1 and BMS mobilize anti-tumor T cell immunity to combat cancer. Reproduced with permission from reference^82^. Copyright 2022 Elsevier. (C) EALP nanoparticles consisted of HSPC, cholesterol, DSPE-mPEG2k, avasimibe, and MMP-2 sensitive peptide-grafted PPa, which accumulated in tumors and released iRGD molecules upon abundant MMP-2. EALP improved T-cell function *via* blocking the cholesterol esterification and induced ICD by PPa-mediated PDT. Reproduced with permission from reference^88^. Copyright 2022 Wiley-VCH.

Wang et al.^81^ proposed an MMP-2-sensitive anti-PD-L1 antibody (aPD-L1)/ICG-based nanoparticle (S-aPD-L1/ICG@NP) that counteracts drug resistance in cancer immunotherapy by blocking the PD-1/PD-L1 cascade (**Figure 3A**). Through enhanced permeability and retention (EPR), aPD-L1/ICG@NP passively accumulates at the tumor site and is activated to release aPD-L1 in high MMP-2-expressing tumors to block PD-L1 on tumor cells. ICG increases intratumoral CTL infiltration and sensitizes tumors to PD-L1 inhibition. As a potent nanoplatform, this system can be used to deliver immune checkpoint inhibitors to overcome drug resistance during cancer immunotherapy. Zhou et al.^82^ developed a bispecific nanomodulator (IQS) to trigger ICD and dual blockade of the PD-L1 and IDO-1 pathways, thus enhancing effective antitumor immunity (**Figure 3B**). IQS is self-assembled from ICG, the BRD4 inhibitor, JQ1, and the IDO-1 inhibitor, BMS986205 (BMS). PTT and ICD are effectively induced under NIR irradiation. Surprisingly, IQS reverses PTT-mediated PD-L1 upregulation and IDO-1 activation, resulting in increased CTL invasion. Furthermore, IQS causes immunologic memory effects that significantly suppress tumor growth. In addition to PD-1/PD-L1, the immunologic checkpoint, CD47, which is expressed on numerous malignant tumor cells, operates as a “do not eat me” signal to block DCs from phagocytosing tumor cells^83^. Monoclonal anti-CD47 antibodies (aCD47) have been tested in clinical trials and have shown great potential^84^. Zhou et al.^85^ designed a TME-activated vesicle for co-delivery of the OXA prodrug and PEGylated photosensitizer (PS) to trigger ICD in tumor cells. The platform showed that the combined effect of prodrug vesicle-induced ICD and aCD47-mediated CD47 blockage boosts anti-tumor immunity of the ICD inducer. Given that CD47 is overexpressed in a variety of tumor cells, ICD plus aCD47 may be a potential strategy for overcoming ICD inducer resistance in cancer immunotherapy.

#### Combining ICD inducers with cholesterol metabolism regulators

It has been reported that elevated cholesterol levels are a common drug resistance phenomenon, and that elevated cholesterol mediates nuclear translocation of the transcription factor, SP1, which binds directly to the estrogen-related receptor alpha (ERRα) promoter. By activating the EGFR/Src/Erk signaling axis, transcription is allowed to occur in the presence of EGFR-TKIs, thus resulting in acquired resistance to EGFR-TKIs^86^. In addition, the development of immunosuppressive metabolites in the TME impairs cholesterol metabolism in tumor-infiltrating CD8^+^ T cells^87^. As a result, inhibiting tumor cell cholesterogenesis is a viable strategy for overcoming immunotherapy resistance. Liu et al.^88^ used lipid nanovesicles co-encapsulated with the MMP-2 enzyme-responsive proinfiltration peptide, iRGD-modified Ppa, and the cholesterol esterase (ACAT) inhibitor, avasimibe, to modulate cholesterol metabolic pathways and enhance PDT (**Figure 3C**). Nanoparticles passively accumulate at the tumor site, and in response to MMP-2, iRGD is released from the nanoparticles, promoting deep tumor penetration of avasimibe. Cholesterol metabolism is critical for T cell signaling and effects^89^. Avasimibe has the potential to inhibit cholesterol metabolism in CD8^+^ T cells and tumor cells, restoring T cell function. Furthermore, PDT-triggered ICD is enhanced by regulation of cholesterol metabolism. This study described a novel approach to improve the efficacy of ICD by modulating cholesterol metabolic pathways.

### SDDSs reverse immunosuppressive TMEs

#### SDDSs targeting immunosuppressive cells

**Figure 4:**
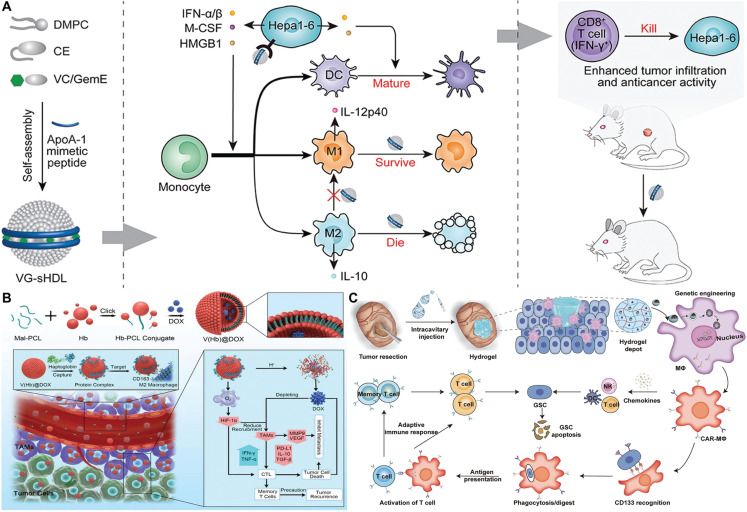
SDDSs targeting immunosuppressive cells. (A) Schematic illustration of the design and mechanism of action of sHDL. Gemcitabine (GemE) and vadimezan (VC) delivered *via* sHDLs promoted monocyte aggregation and differentiation toward M1, while also killing M2, resulting in elevated intratumoral IL-12p40 and decreased IL-10. Increased DC-mediated antigen expression and recruitment of CD8^+^ T cells resulted in potent antitumor effects. Reproduced with permission from reference^90^. Copyright 2021 Elsevier. (B) Schematic illustration of engineered endogenous TAM-targeted biomimetic nano-RBC reprogrammed with TME to enhance chemo-immunotherapy for cancer. Reproduced with permission from reference^91^. Copyright 2021 Wiley-VCH. (C) Schematic illustration of the local production of CD133-specific CAR-MΦs around the tumor cavity by intratumoral injection of NP-hydrogel superstructure for preventing postoperative GBM relapse. NK, natural killer; and DC, dendritic cell. Reproduced with permission from reference^92^. Copyright 2022 American Association for the Advancement of Science.

Targeting immunosuppressive cells in the TME, such as MDSCs and TAMs, to restore anti-tumor immunity by inhibiting immunosuppressive cell function is thought to be a promising strategy for overcoming drug resistance in cancer immunotherapy. Antagonism of immunosuppressive cells on T-cell function can be decreased, boosting ICB potency; however, such antagonism can minimize the production of aberrant metabolites in the TME and cause drug inactivation. Wang et al.^90^ created a synthetic high-density lipoprotein (sHDL) to regulate the interactions of intratumoral M2 macrophages, DCs, and MDSCs (**Figure 4A**). Vadimezan, an agonist of the mouse interferon gene stimulator, STING, and/or gemcitabine (Gem), a DNA synthesis inhibitor, were loaded into the sHDLs. Gem selectively kills M2 macrophages in sHDLs, whereas vadimezan promoted DC maturation and monocyte differentiation into antitumor M1 macrophages. These sHDLs effectively reverse M2-mediated immunosuppression, decrease MDSC differentiation, and increase the percentage of M1 macrophages, thereby significantly improving immunotherapeutic efficacy. Furthermore, using a biomimetic delivery system to target immunosuppressive cells can effectively evade immune system clearance, while allowing active targeting of drugs to specific cells for precise delivery. Wang et al.^91^ created a self-assembled nanorethrocyte system [V(Hb)] for delivery of the chemotherapeutic, adriamycin (DOX), to reprogram TAMs (**Figure 4B**). For effective cell killing, the Hb fraction binds to endogenous plasma haptoglobin (Hp) and specifically targets M2-type TAMs *via* CD163 surface receptors. TAM targeting effectively reduces levels of immunosuppressive cytokines (IL-10 and TGF-β), while increasing immunostimulatory IFN-γ and CTL anti-tumor killing responses. This endogenous TAM-targeting biomimetic system is a promising tool that can be used in conjunction with ICB to combat drug resistance in cancer immunotherapy.

In addition to direct drug delivery to immunosuppressive cells, *in situ* genetic engineering of immunosuppressive cells in the TME has been developed as a novel strategy for overcoming drug resistance in cancer immunotherapy. This engineering strategy transforms immunosuppressive cells into anti-tumor subtypes, resulting in long-lasting tumor killing effects. Chen et al.^92^ developed a nanoparticle-hydrogel capable of generating glioma stem cell (GSC)-specific chimeric antigen receptor macrophages/microglia (CAR-MΦs) around the lumen of postoperative glioma lesions (**Figure 4C**). After intraluminal delivery, nanoparticles transfer CAR genes into broadly-distributed M2-type macrophages, resulting in CAR-MΦs in a glioma animal model. CAR-MΦs exhibit M1-type macrophage features and are capable of seeking out and engulfing GSCs as well as clearing remaining GSCs in TME by triggering adaptive anti-tumor immune responses. When combined with the aCD47 antibody, the number of positive immune response cells increases.

#### SDDSs targeting extracellular matrix (ECM) components and stromal cells

**Figure 5:**
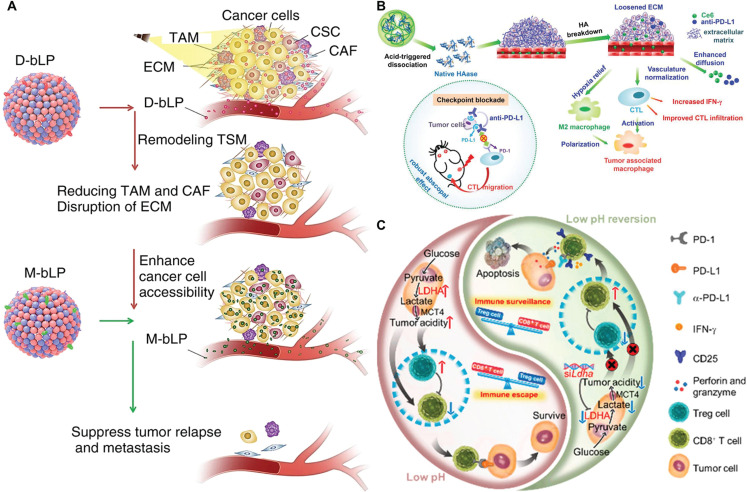
SDDSs targeting ECM components and acidic and hypoxic TME. (A) The bioinspired lipoprotein (bLP) nanosystem loaded with DiR photothermal agent (D-bLP) and anti-cancer drug mertansine (M-bLP), respectively, to remodel the tumor stromal microenvironment (TSM) barrier through D-bLP-mediated photothermal action and enhance the accumulation of second M-bLP in tumor, thereby effectively suppress tumor relapse and metastasis. Reproduced with permission from reference^95^. Copyright 2019 Springer Nature. (B) pH-responsive dextran (DEX)-hyaluronidase (HAase) polymer (DEX-HAase) nanoparticles induced the breakdown of cross-linked hyaluronic acid (HA) and, in combination with PD-L1 checkpoint blockers, enhanced PDT and antitumor immune responses. Reproduced with permission from reference^96^. Copyright 2019 WILEY-VCH. (C) Vesicular nanoparticle (VNP) system to deliver si*Ldha* to tumor sites to knock down lactate dehydrogenase. VNP reversed tumor immunosuppressive microenvironment through tumor acidity modulation. Reproduced with permission from reference^99^. Copyright 2019 American Chemical Society.

The aberrant deposition of ECM outside tumor cells increases the pressure inside the solid tumor, thus reducing the ability of nanomedicines and immune cells to penetrate deep into tumors, which leads to drug resistance^93,94^. Treatment techniques targeting the ECM typically involve matrix-degrading enzymes to hydrolyze the excess protein matrix or functional ablation of the ECM with suitable medicines. Tan et al.^95^ reported a bioinspired lipoprotein (bLP) that induces efficient PTT to remodel the ECM (**Figure 5A**). Multiple stromal cells and ECM components of the TME are extensively disrupted following bLP-mediated PTT, resulting in a 4.27-fold increase in the secondary accumulation of bLP in tumors, deep penetration throughout the tumor block, and a 27.0-fold increase in cancer cell accessibility. Notably, ECM remodeling greatly suppressed tumor development and resulted in a 97.4% inhibition of lung metastasis. Wang et al.^96^ constructed a pH-responsive dextran (DEX)-hyaluronidase (HAase) polymer (DEX-HAase) nanoparticle (**Figure 5B**). DEX-HAase dissociates within the acidic TME, releasing native HAase, which induces the breakdown of HA and weakens the ECM structure, allowing oxygen and other therapeutic agents to penetrate more effectively. After pretreatment with DEX-HAase, the therapeutic response to PDT combined with aPD-L1 is greatly improved. This study presented a new adjuvant nanodrug that disrupts the ECM to enhance PDT immunotherapy. Although ECM degradation improves the permeability of nanomedicines and immune cells, ECM degradation also facilitates tumor cell extravasation into blood vessels, resulting in tumor metastasis and recurrence^93^. As a result, how to minimize tumor cell extravasation is critical to consider in ECM-targeted therapy. Liu et al.^97^ developed a self-assembled liposome that was subsequently conjugated with anti-fibroblast (CAF) antibodies to ensure that prolyl isomerase Pin1 inhibitors are specifically delivered to CAFs. This technique efficiently reduces CAF function while causing less ECM damage and represents an alternative strategy for overcoming the stromal barrier in pancreatic cancer.

#### SDDSs targeting the tumor metabolism microenvironment

The excessively active growth of tumors creates a number of abnormal metabolic microenvironments, such as abnormal elevation of glycolipid metabolism and accumulation of the glycolytic product, lactate, thus generating an acidic environment (pH = 6.5–6.8), internal hypoxia, upregulation of ROS levels, and overexpression of protease levels. Among these effects, the creation of an acidic and hypoxic TME not only promotes tumor growth, but also significantly inhibits the efficacy of chemotherapeutic drugs and photosensitizers, which may be inactivated by structural alterations in the acidic environment. Current SDDS-based management of an acidic tumor environment is mostly accomplished through the following: (1) depletion of lactate in the TME; and (2) suppression of lactate synthesis in tumors. Modification of the hypoxic environment of the tumor primarily consists of the following: (1) the *in situ* creation of oxygen by catalyzing excess H_2_O_2_ in the TME; and (2) direct supply of oxygen *via* oxygen carriers.

Zhu et al.^98^ developed CaCO_3_ nanoparticles (DNCaNPs) for co-encapsulating DOX and alkylated NLG919 (aNLG919). CaCO_3_ permits therapeutic medicine to enter deep into a tumor and effectively neutralizes the acidic tumor pH, thus facilitating immunosuppression reversal. Zhang et al.^99^ used a vesicular nanoparticle (VNP) system to deliver siLdha to tumor sites to knock down lactate dehydrogenase (LDHA), which plays a key role in lactate production, to reduce lactate production, reverse the acidic microenvironment of tumors, restore T cell anti-tumor function, and enhance the therapeutic effect of the immune checkpoint inhibitor, PD-1 antibody (**Figure 5C**). Prasad et al.^100^ developed a bioinorganic nanoparticle composed of polyelectrolyte albumin complexes and manganese dioxide (MnO_2_ NPs) to synthetically regulate oxygen depletion, acidity, and ROS in the TME. MnO_2_ NPs catalyze the synthesis of O_2_ from H_2_O_2_ and neutralize acidic pH, thus improving the oxygen-depleted and acidic environment of tumors and increasing the sensitivity of oxygen-dependent radiotherapy. Hemoglobin in erythrocytes is an ideal oxygen-conducting material because it is a natural *in vivo* oxygen delivery protein. Tang et al.^101^ directly attached activated erythrocytes to nanoparticles loaded with photosensitizers to improve the therapeutic effect of photosensitizers using the oxygen supply capacity of erythrocytes, and effectively inhibited the growth of malignant glioblastomas.

### SDDSs regulating type I IFN and PTEN signaling pathways

**Figure 6:**
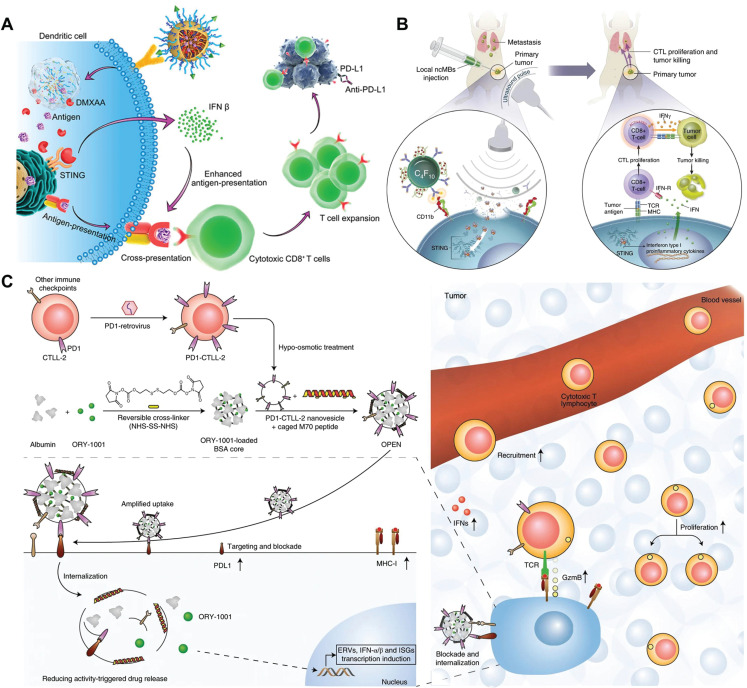
SDDSs regulating the IFN signaling pathway. (A) Acid-responsive polymer nanovaccine boosting the STING pathway and augmenting the T-cell immune response for improved cancer immunotherapy. Reproduced with permission from reference^110^. Copyright 2020 American Chemical Society. (B) Ultrasonic (US)-guided cancer immunotherapy platform to manipulate STING activation and downstream antitumor immunity. Reproduced with permission from reference^111^. Copyright 2022 Springer Nature. (C) Epigenetic nanoinducer (OPEN) to enter PDL1-expressing cells and upregulate the expression of IFNs, MHC-I, and PDL1. The upregulated PDL1 is neutralized by subsequent OPEN, which enhanced the recruitment, proliferation, and activity of CTLs in the tumor. M70, macrolittin 70; ERV, endogenous retroviruse; TCR, T cell receptor; GzmB, granzyme B. Reproduced with permission from reference^115^. Copyright 2021 Springer Nature.

Tumor cell intrinsic factors that contribute to immunotherapy resistance include the expression or inhibition of genes and pathways in tumor cells that prevent immune cell infiltration or function within the TEM^25^. IFN improves immune function by increasing MHC-I expression^102^. Therefore, IFN has a role in antigen detection and the interactions between adaptive and innate immune cells. To this end, loss-of-function mutations and genetic changes in the IFN signaling system are linked to clinical drug resistance in immunotherapy^103,104^. Recently, STING has been linked to anti-infection, anti-inflammatory, and anti-tumor therapies^105^. Activation of the STING signaling pathway in APCs causes cellular production of IFN-I, which triggers a T cell-mediated adaptive immunologic response. Natural STING agonists, such as cyclic GMP-AMP (cGAMP), have a weak entrance retention capacity. Furthermore, non-specific activation of STING may result in a wide inflammatory response.^105^ SDDSs enable the targeted delivery of STING agonists of APCs to attenuate the negative effects, while overcoming immunotherapy resistance in malignancies due to decreased IFN production^106–109^. Zhou et al.^110^ demonstrated an acid-responsive polymer nanovaccine that activates the STING pathway (**Figure 6A**). The nanovaccine contains a STING agonist and a neoantigen that accumulates in lymph nodes and promotes DC uptake. The STING agonist stimulates the STING pathway in DCs, increases IFN-β production, and enhances T cell activation. When combined with an anti-PD-L1 antibody, STING increased antitumor activity in a 4T1 breast tumor model, implying that activation of the STING pathway eliminated drug resistance to ICB therapy. To manipulate STING activation, Li et al.^111^ created an ultrasonic (US)-guided cancer immunotherapy platform using nanocomplexes made of cGAMP electrostatically coupled to microbubbles targeting APCs (**Figure 6B**). The nanocomplexes are passively deposited in LNs, and the linked microbubbles attached to APCs are used to efficiently transfer cGAMP to the cytoplasm *via* ultrasound, which triggers the cGAS-STING signaling pathway and effectively initiates an antigen-specific T cell immune response. This process not only provides a perfect platform for STING activation under controlled conditions, but also advances imaging-guided cancer immunotherapy. Furthermore, STING agonists can be targeted and administered to tumor tissues to generate a local immune response. Dane et al.^112^ used cleavable linkers to attach STING-activated cyclic dinucleotides (CDNs) to PEGylated lipids and incorporated the lipids into disk-shaped nanoparticles (LND-CDNs). LND-CDNs penetrate tumors more effectively than liposomes and expose tumor cells to STING agonists. The uptake of LND-CDNs by tumor cells increases co-localization of CDNs and tumor antigens in DCs, resulting in significant T-cell activation.

Cells must have functional STING proteins for cGAMP to initiate STING signaling. It has been demonstrated that epigenetic silencing of STING or cyclic guanosine monophosphate (GMP)-adenosine monophosphate (AMP) synthase (cGAS) impairs STING signaling in cancer cells^113^. In addition, IFN expression causes the activation of several immunologic checkpoints, resulting in immune evasion^114^. Zhai et al.^115^ developed an epigenetic nanoinducer (OPEN) loaded with ORY-1001 (a selective lysine-specific histone demethylase 1 inhibitor) and overexpressing PD-1 membrane-modified T cells to overcome these challenges (**Figure 6C**). The OPEN identified PD-L1 on tumor cells and internalized it, upregulating intra-tumor IFN, membrane MHC-I, and membrane PD-L1 expression. MHC-I facilitated neoantigen presentation. In addition, PD-L1 overexpression increased the tumor-specific accumulation and absorption of subsequent OPEN, which inhibited PD-L1 expression. Complementing intratumoral IFNs by modulating epigenetics and limiting the immunosuppressive function improves CTL recruitment, proliferation, and activity, with significant anticancer efficacy in numerous tumor types.

The collective clinical evidence has demonstrated that the deletion or mutation of tumor suppressor genes may be associated with the TME and poor response or resistance to ICB therapy. Deletion of the PTEN gene, a protein that negatively regulates the PI3K-AKT pathway, has been shown to be associated with decreased T-cell infiltration and immunosuppressive cell proliferation^116,117^. Restoration of PTEN expression by plasmid DNA transfection of tumor cells can reverse immunotherapy resistance^118^; however, systemic instability has hindered the clinical application of plasmid DNA. To overcome these issues, Islam et al.^119^ used polymer-lipid hybrid nanoparticles for the systemic delivery of modified *PTEN* mRNA to tumor cells, which effectively overcame several potential challenges in mRNA delivery, including large size, high negative charge, easy degradation, and poor protein translation. These carriers can enter cancer cells, protect mRNA from degradation, and restore natural immune function *in vivo*. PTEN deficiency promotes resistance to T cell-mediated immunotherapy, suggesting that restoration of PTEN function by *PTEN* mRNA nanoparticle technology may be useful in the application of immunotherapy and in rescuing drug sensitivity in immunotherapy-resistant tumors^120^. Liu et al.^121^ further designed ApoE-modified pH-responsive mRNA nanoparticles (ABNPs@mRNA) coated with red blood cell membranes as a non-invasive brain delivery system for selective delivery of *PTEN* mRNA across the blood-brain barrier to treat glioblastomas.

### SDDSs enhancing ACT efficacy

**Figure 7:**
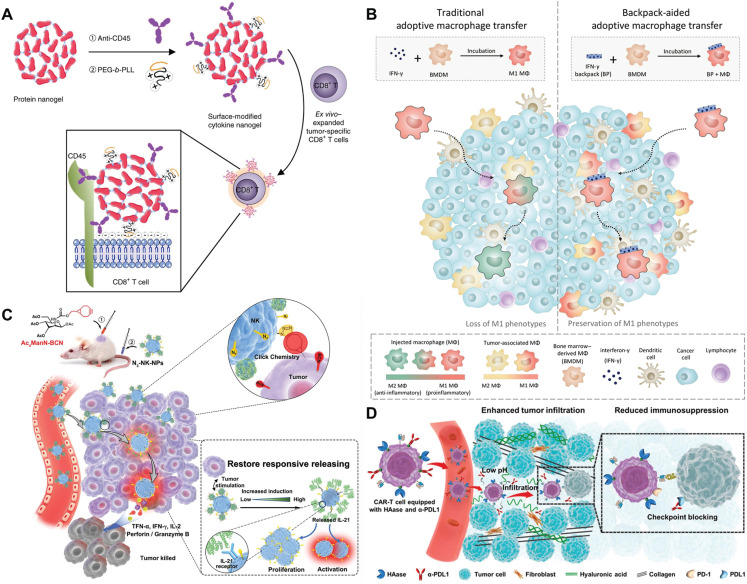
SDDSs enhancing the efficacy of ACT. (A) Protein nanogel (NG) capable of enhancing T cell activation by “packaging” a large number of supportive protein drugs on T cells and releasing them selectively upon T-cell receptor activation. Reproduced with permission from reference^128^. Copyright 2018 Springer Nature. (B) Unlike MΦs polarized with IFN-γ *ex vivo* quickly shifting from a pro-inflammatory phenotype to an anti-inflammatory phenotype after penetrating a solid tumor, MΦs carrying IFN-γ–loaded backpacks maintain pro-inflammatory phenotypes and regulate the phenotype of endogenous TAMs deep in the tumor microenvironment. Reproduced with permission from reference^130^. Copyright 2020 American Association for the Advancement of Science. (C) Live-cell nanocarriers N_3_-NK-NPs based on bio-orthogonal metabolic glycol-engineering and hitchhiker delivery system effectively promoted NK cytotoxicity, infiltration, and homing to tumors *in vivo*, thereby enhancing anti-tumor efficacy. Reproduced with permission from reference^131^. Copyright 2022 Wiley-VCH. (D) CAR-T cells conjugated with HAase and aPDL1 antibodies to increase CAR-T therapeutic efficacy against solid tumors. Reproduced with permission from reference^133^. Copyright 2022 American Chemical Society.

In contrast to other types of immunotherapies, ACT directly induces a robust antigen-specific immune response *via* the delivery of immune cells generated and expanded *in vitro*^122^. In addition to CAR-T, which has been approved by the United States Food and Drug Administration (FDA) for the treatment of hematomas, NK cell-, DC-, and macrophage-based ACT have been developed. Immune cells have a longer half-life and systemic dispersion than small chemicals and biologics, and have the potential to overcome physiologic boundaries and recognize and respond to pathogenic stimuli^123^. These benefits make immune cells promising cancer-fighting weapons; however, bacause of the immunosuppressive TME in tumor tissues, it is difficult for immune cells to move efficiently to the lesion location^68,124^. The few immune cells that reach the tumor are likewise vulnerable to failure and death as a result of local immunosuppressive signals^122,125^. This resistance to immune cell therapy due to intrinsic and extrinsic tumor environmental factors has limited the clinical application of ACT. By attaching to diverse biofunctional molecules, SDDSs avoid drug resistance to ACT^126^. Nanoparticles and antibodies have high structural adaptability and can enable multifunctionalization of immune cells *via* cell surface conjugation, giving immune cells increased efficacy or the ability to bypass immunosuppressive barriers^127^. Tang et al.^128^ reported that activating TCR signaling increases the reduction potential on the surface of T cells and produced a protein nanogel (NG) capable of responding to reduction potential changes (**Figure 7A**). The NGs are comprised of therapeutic protein molecules connected together by a disulfide-containing bis-N-hydroxy succinimide (NHS) linker. Anchoring NGs to CD45 on T cells prevents endocytosis by the cells. Using an IL-15 super agonist combination (IL-15Sa) as the test medication, T cells equipped with NG backpack were able to rapidly grow within the tumor (16 times more than systemic IL-15Sa treatment) but remained quiescent in the peripheral circulation. Hao et al.^129^ also used hydrophobic forces to bind lipids to the T cell membrane before coupling the complex to avasimibe-encapsulated liposomes *via* a click reaction on the T cell membrane. Avasimibe was restricted to the T cell surface throughout T cell circulation and extravasation, then released locally to increase the cholesterol concentration in the T cell membrane, promote fast aggregation of T cell receptors, and sustain T cell activation.

In addition to T cells, nanoparticles can modify macrophages and NK cells to provide multivalent activities. Shields et al.^130^ created a disk-like particle that adheres to the surface of macrophages and modulates the phenotype (**Figure 7B**). The backpack is comprised of two layers of polylactic acid-hydroxyacetic acid co-polymer (PLGA) with polyvinyl alcohol (PVA) and IFN-γ. Owing to the form effects, macrophages do not endocytose the backpack. Macrophages carrying the IFN backpack remain in M1 for an extended period of time, preserving the phenotype within immunosuppressive tumors and increasing anti-tumor activity. Meng et al.^131^ constructed an NK cell backpack (N_3_-NK-NPs; **Figure 7C**). Complementary bioorthogonal groups {azide (N_3_)/bicyclo[6.1.0]nonyl} (BCN) were inserted into NK cells (N_3_-NK) and tumor cells (BCN-Raji). IL-12-containing nanoparticles (ILNPs) were conjugated to the surfaces of NK cells using aCD45 antibodies. The bioorthogonal method successfully promoted NK cell tumor targeting. Sustained IL-12 release activated NK cells and significantly increased proliferation and activation, hus timproving the therapeutic potential. Furthermore, IL-21 induced the recruitment of numerous immune cells, thereby activating the innate immune system. The fundamental mechanism underlying NK cell-mediated tumor cell death is the creation of an immunosynaptic (IS) detection of cancer cells. In light of this, Im et al.^132^ developed a smart DOX-releasing NK cell for solid tumor immunotherapy. NK cells were modified with IS-responsive drug-loaded micelles, and upon IS formation, the micelles disintegrated to release DOX with the production of acidic granule contents, effectively increasing NK cell tumor death. These “backpack” techniques successfully improve the function of immune cells, increasing the potential applicability.

Moreover, backpacks provide immune cells with the ability to bypass immunosuppressive obstacles. Zhao et al.^133^ created CAR-T cells in combination with HAase and aPD-L1 antibodies to increase therapeutic efficacy against solid tumors (**Figure 7D**). The modified HAase destroys hyaluronic acid and damages the tumor ECM, allowing CAR-T cells to enter solid tumors. Furthermore, the aPD-L1 antibody significantly suppresses PD-1 on the tumor surface, preventing drug resistance in cancer immunotherapy. This approach is expected to address issues, such as CAR-T solid tumor invasion rates.

## Conclusions

In this review we have discussed how SDDSs can overcome drug resistance in cancer immunotherapy. Multiple therapeutic compounds can be selectively administered to tumor cells or immune and stromal cells in the TME using SDDSs to combat drug resistance and promote anti-cancer immunotherapy by activating ICD in tumor cells, reversing tumor immunosuppression in the TME, or altering IFN signaling pathways. In addition, SDDSs combined with ACT provide a better therapeutic effect and have the ability to overcome immunosuppressive barriers in immune cells, effectively overcoming drug resistance to ACT.

Effective SDDS-based techniques for overcoming drug resistance and improving cancer immunotherapy are emerging; however, significant difficulties remain in achieving good therapeutic effects and practical applications. First, some SDDSs currently being developed have non-biodegradable components that may induce toxicity in the organisms. The intrinsic immunomodulatory potential of certain materials used to produce SDDSs may result in excessive inflammatory responses or drug resistance. Therefore, one of the keys to the clinical translation of SDDSs is the creation of biocompatible and biodegradable materials. Furthermore, the use of diverse bio-derived vesicles as drug delivery carriers has considerably reduced the possible negative effects of SDDSs. Second, while most recent studies have shown SDDSs to be effective in mouse models, there is no certainty that SDDSs would be effective in human cancers owing to species differences. Most contemporary laboratory models are subcutaneous tumor models, and the tumor cells used in the laboratory are cell lines with well-defined phenotypes that differ dramatically from tumors produced in the natural environment. Therefore, future studies are needed to build *in situ* or various forms of factor-induced tumor models, as well as the use of humanized tumor cells or humanized mice that can better replicate the process of tumor treatment in clinical settings. Finally, SDDSs will need to address the obstacle of drug resistance by more than simply encapsulating therapeutic medications in delivery vehicles to be taken up by tumor cells; instead, employing SDDSs to preferentially transport drugs to immune cells appears to be a more viable approach. Although immune cells have an important role against cancer in all stages of cancer formation, progression, and treatment, different drug resistance mechanisms are fundamentally an adaptive development of cancer cells. The use of SDDSs to modify or equip immune cells not only improves immune cell function but also strategically realizes the all-around effect of the immune system against cancer. Overall, future novel SDDSs for overcoming drug resistance in cancer immunotherapy will undoubtedly widen the applicability of immunotherapy in solid tumors.
